# Effectiveness of phototherapy incorporated into an exercise program for osteoarthritis of the knee: study protocol for a randomized controlled trial

**DOI:** 10.1186/1745-6215-15-221

**Published:** 2014-06-11

**Authors:** Carly de Faria Coelho, Ernesto Cesar Pinto Leal-Junior, Daniela Aparecida Biasotto-Gonzalez, André Serra Bley, Paulo de Tarso de Camillo de Carvalho, Fabiano Politti, Tabajara de Oliveira Gonzalez, Adriano Rodrigues de Oliveira, Marcelo Frigero, Marilia Barbosa Santos Garcia, Almir Vieira Dibai-Filho, Cid André Fidelis de Paula Gomes

**Affiliations:** 1 Postgraduate Program in Biophotonics Applied to Health Sciences, Universidade Nove de Julho (UNINOVE), Av. Dr. Adolfo Pinto,109, Água Branca, 05001-100 São Paulo, SP, Brazil; 2 Postgraduate Program in Rehabilitation Sciences, Universidade Nove de Julho (UNINOVE), Av. Dr. Adolfo Pinto,109, Água Branca, 05001-100 São Paulo, SP, Brazil; 3 Department of Physical Therapy, Universidade Nove de Julho (UNINOVE), Av. Dr. Adolfo Pinto,109, Água Branca, 05001-100 São Paulo, SP, Brazil; 4 Postgraduate Program in Rehabilitation and Functional Performance, University of São Paulo, Av. Bandeirantes, 3900, Curso de Fisioterapia, Monte Alegre, 14049-900 Ribeirão Preto, SP, Brazil

**Keywords:** Phototherapy, Osteoarthritis of the knee, Postural balance, Lower limb strength, Physical therapy modalities

## Abstract

**Background:**

Osteoarthritis is a chronic disease with a multifactor etiology involving changes in bone alignment, cartilage, and other structures necessary to joint stability. There is a need to investigate therapeutic resources that combine different wavelengths as well as different light sources (low-level laser therapy and light-emitting diode therapy) in the same apparatus for the treatment of osteoarthritis. The aim of the proposed study is to analyze the effect of the incorporation of phototherapy into a therapeutic exercise program for individuals with osteoarthritis of the knee.

**Methods/Design:**

A double-blind, controlled, randomized clinical trial will be conducted involving patients with osteoarthritis of the knee. Evaluations will be performed using functional questionnaires before and after the treatment protocols, in a reserved room with only the evaluator and participant present, and no time constraints placed on the answers or evaluations. The following functional tests will also be performed: stabilometry (balance assessment), dynamometry (muscle strength of gluteus medius and quadriceps), algometry (pain threshold), fleximeter (range of motion), timed up-and-go test (functional mobility), and the functional reach test. The participants will then be allocated to three groups through a randomization process using opaque envelopes: exercise program, exercise program + phototherapy, or exercise program + placebo phototherapy, all of which will last for eight weeks.

**Discussion:**

The purpose of this randomized clinical trial is to analyze the effect of the incorporation of phototherapy into a therapeutic exercise program for osteoarthritis of the knee. The study will support the practice based on evidence to the use of phototherapy in individuals with a diagnosis of osteoarthritis of the knee. Data will be published after the study is completed.

**Trial registration:**

The protocol for this study has been submitted to Clinical Trials, registration number NCT02102347, on 29 March 2014.

## Background

Osteoarthritis (OA) is a chronic condition of the joints, the most commonly affected of which is the knee. This condition is characterized by joint pain and in more advanced stages, joint deformities, contracture, and muscle atrophy leading to severe disability. Poor bone alignment, ligament injuries, and meniscal tears that lead to cartilage wear due to excessive impact and joint instability seem to be strongly correlated with the origin of this condition [1,2]. These factors, together with the increase in pain and diminished function, create difficulties in executing functional activities and can compromise postural stability, leading to a greater risk of falls [3].

Non-steroidal anti-inflammatory drugs (NSAIDs) are the first line of treatment in cases of OA to relieve pain and inflammation [1]. More recently, chondroitin and glucosamine have been used, which are essential components to cartilage formation but have not yet demonstrated consistent results regarding pain reduction and slowing down the narrowing of the joint space [4]. Indeed, drug therapies have proven insufficient in achieving the required results [5,6].

The combination of medicinal and non-medicinal treatment is essential to the maintenance of functional autonomy of movement [7]. Active exercises combined with muscle strengthening and stretching as well as improvements in physical fitness through aerobic exercise [7,8] are the main treatment options for patients with OA of the knee.

Low-level laser therapy (LLLT) has properties that exert a positive influence on the proliferation of fibroblasts, collagen synthesis, and bone regeneration. LLLT also has an analgesic effect mediated by hormonal and opioid mechanisms and has therefore recently become an important tool in the treatment of OA, offering pain relief and improved knee function [9,10]. The use of phototherapy in the form of LLLT and light-emitting diode therapy, both of which operate in the red and infrared spectra, has proven effective in delaying the development of fatigue and improving muscle performance [11]. It would therefore seem advantageous to combine these two forms of phototherapy in a single device. However, clinical trials are needed to test the effects on deeper structures, such as joints, using comparable protocols and outcome measures. Due to its physiological effects, phototherapy may promote increased positive effects of preexisting treatments and may slow the degeneration process and its repercussions, such as contractures and joint deformity, which can lead to postural imbalance and the loss of muscle strength in the lower limbs, resulting in recurrent falls.

The aim of the proposed study is to analyze the effect of the incorporation of phototherapy into a therapeutic exercise program for OA of the knee. The hypothesis is that patients with OA of the knee who are treated with phototherapy combined with a therapeutic exercise program will experience a greater reduction in pain as well as improved knee range of motion, static balance, functional capacity, muscle strength, and quality of life in comparison with those treated with exercise alone.

## Methods/Design

### Overview of research design

We propose a double-blind, placebo-controlled, randomized clinical trial to analyze the effect of the incorporation of phototherapy into a therapeutic exercise program for individuals with osteoarthritis of the knee. Participants will be recruited from clinics and hospitals and will be allocated to different groups through a randomization process using opaque envelopes containing cards stipulating one of the three following groups: 1) exercise program, 2) exercise program + phototherapy, and 3) exercise program + placebo phototherapy. Phototherapy will be performed on the knees diagnosed with OA.

The study will be divided into two evaluations and one treatment phase, to which all individuals will be submitted. Phase one is the initial evaluation phase. After recruitment, individuals who meet the eligibility criteria will undergo an initial evaluation in the following sequence: administration of the Lower Extremity Functional Scale (LEFS), the WHOQOL-BREF questionnaire and the Western Ontario and McMaster Universities Index for Osteoarthritis (WOMAC), numeric pain rating scale (NPRS) and algometry for pain, followed by a functional evaluation (stabilometry, dynamometry, range of motion (ROM), timed up-and-go test (TUGT) and the functional reach test (FRT)). Phase two is the treatment phase. Following randomization, the treatments will be performed on the knee(s) in three weekly sessions spanning eight weeks (for a total of 24 sessions). Phase 3 is the final evaluation phase. One day after the end of the treatment phase, the final evaluation will be performed, following the same sequence employed in the initial evaluation (Additional file 1).

### Blinding

The participants in exercise program + phototherapy and exercise program + placebo phototherapy will be blinded to the form of treatment (active or placebo phototherapy). The researcher in charge of the randomization process will program the phototherapy device based on the randomization results and will then turn the device over to another researcher for application. Three other researchers blinded to the phototherapy protocol will individually be in charge of the procedures in each group. These researchers will not participate in the evaluation phases. The evaluations will be carried out by a fourth researcher blinded to the allocation of the participants to the different groups. The statistician will be blinded to the allocation and procedures until the conclusion of the statistical analysis.

### Inclusion criteria

The inclusion criteria are as follows: diagnosis of OA of the knee (bilateral) confirmed by the participant’s physician according to criteria of the American College of Rheumatology; Grades 2 to 4 on the Kellgren-Lawrence OA scale (0 = absence of OA, 1 = doubtful, 2 = minimal, 3 = moderate, and 4 = severe); aged between 40 and 80 years; either gender; and knee pain and functional disability for at least three months, as confirmed by the medical history of each patient. The participants will be allowed to use medications prescribed by their doctor. The use of analgesics or anti-inflammatory drugs will be monitored throughout the study. The name, dosage, and frequency of the medications will be recorded and presented in a table.

### Exclusion criteria

The exclusion criteria are as follows: a history of trauma to the knees; cognitive or psychological disorder; neurological disorder (sensory or motor); cancer; diabetes; acute adverse health condition; symptoms of OA of the hip; cardiopulmonary disease that impedes walking; need for a wheelchair or gait-assistance device; received steroids through intra-articular injection or orally in the previous six months; or received physical therapy treatment for the knee within last four weeks. The exclusion criteria will be determined by access to the medical history of individuals.

### Ethics and data security

This trial received approval from the Human Research Ethics Committee of University Nove de Julho (Brazil) under process number 525.849, dated 10 February 2014. All individuals will be asked to provide written informed consent prior to randomization using a standard form. This trail is registered with the World Health Organization at ClinicalTrials.gov under number NCT02102347 on 29 March 2014.

### Procedures

#### Questionnaires and scales

The following questionnaires and scales will be administered before and after the treatment protocols: Lower Extremity Functional Scale: Scale consisting of 20 items developed to evaluate musculoskeletal dysfunction in the lower limbs [12]. The scores for each item are 0 (extreme difficulty or unable to perform the activity), 1 (quite a bit of difficulty), 2 (moderate difficulty), 3 (a little bit of difficulty), and 4 (no difficulty). The maximum score is 80 points [13]. Numeric pain rating scale: A simple, easily administered scale made up of a set of numbers ranging from 0 (absence of pain) to 10 (worse pain imaginable) [14]. The degree of pain in the knee(s) with OA will be measured with the participant at rest, before the use the algometry. Quality of life will be evaluated using the WHOQOL-BREF, which is a short version of the WHOQOL-100 developed by the Quality of Life Group of the World Health Organization. The WHOQOL-BREF is made up of 26 items: two addressing overall health and the others divided among four subscales (physical health, psychological health, social relations, and environment). Each item is scored based on a Likert scale ranging from 1 to 5 points. At the end of the study, the results will be transformed into a linear scale ranging from 0 to 100, with higher scores denoting a better quality of life [15,16]. Besides being administered during the initial and final evaluations, the WHOQOL-BREF will also be administered three and six months after the final evaluation through telephone contact.The WOMAC is a specific index for the evaluation of pain, stiffness, and physical function in individuals with OA of the knee and/or hip. This index offers separate scores for the different subscales and contains 24 items: five on pain (score: 0 to 20); two on stiffness (score: 0 to 8), and 17 on physical functioning (score: 0 to 68) [17]. Higher scores indicate worse pain, stiffness, and functional limitation. All questionnaires and scales will be administered in a private room and the participants will be instructed to fill out all items. If a participant refuses or is incapable of cooperating, the researcher will mark the exam with the letters ‘NR’, denoting ‘not registered’, and will also write down the reason for the refusal or inability to complete the questionnaire. All assessment tools will be administered without time constraints to avoid the occurrence of rushed responses.

#### Functional tests

The Biomec 400 v1.1™ force plate (EMG System do Brasil (EMG System do Brasil Ltda® - http://www.emgsystem.com.br - Rua Porto Principe, 50 - Vila Rubi CEP12245-572-São José dos Campos/SP. Phone: 55 12 3922-4069/55 12 3942–4736) will be used for the stabilometric analysis. This system quantifies the distribution of vertical ground reaction force in a static or dynamic position with a sampling frequency of 40 Hz using four sensors (two anterior and two posterior) capable of supporting 150 Kg. Each sensor stores analog data, which are amplified, converted into digital data, recorded, and interpreted using the Biomec 400™ software program (EMG System do Brasil (EMG System do Brasil Ltda® - http://www.emgsystem.com.br - Rua Porto Principe, 50 - Vila Rubi CEP12245-572-São José dos Campos/SP. Phone: 55 12 3922-4069/55 12 3942–4736). The distribution of force on the four points and changes in body sway in the anteroposterior (y axis) and mediolateral (x axis) directions will be evaluated using mean displacement and area. The force plate will be placed on a flat surface, which will be verified by levels attached to the extremities of the plate. The participants will be positioned as follows: in quiet, standing, barefoot, feet abducted at 30° and separated by 3 cm, arms alongside the body, and gaze fixed on a round target (5 cm in diameter) at the height of the glabellum positioned on a pedestal 1.5 meters from the participant. Two stabilometric readings will be performed under two different conditions (eyes open and eyes closed). Each reading will last 30 seconds, with a one-minute interval between readings.

A portable dynamometer (Lafayette manual muscle system, model 01163, Lafayette Instrument Company, Lafayette, Indiana, United States) will be used. This device has been broadly employed in clinical practice and scientific procedures due to its good inter-examiner and intra-examiner reliability in comparison to other evaluation measures considered the gold standard for this type of evaluation [18,19]. Four five-second readings will be taken of maximum voluntary isometric contraction (MVIC) with a 30-second rest period between contractions. The participant’s hands will be crossed over the chest. Readings will be made of the quadriceps and gluteus medius, bilaterally. The first MVIC will be to familiarize the participant with the task. If a participant is unable to performed three contractions with less than 10% variability, a new set of readings will be performed. Strong, constant, verbal stimuli will be given throughout the test. The order of the MVIC readings will be randomized to avoid bias. The procedure for the gluteus medius readings will be: Participant lying on one side with the limb to be tested on top in neutral position, maintained using pillows placed between the legs. An adjustable non-elastic strap will be placed over the iliac crest and attached around the cot to stabilize the pelvis. The dynamometer will be positioned over the lateral femoral condyle under a non-elastic strap attached around the distal thigh and cot. MVIC will be solicited in the form of hip abduction [20]. The procedure for the quadriceps readings will be: Participant in the sitting position, hip flexed at 90° and knee flexed at 60°. The dynamometer will be positioned at the level of the malleoli in the anterior region under a non-elastic strap attached around the distal tibia and cot. MVIC will be solicited in the form of knee extension [21]. Strength data, measured in kilograms (Kg), will be normalized by body weight (Kg) using the following formula: Kg (muscle strength)/Kg (body mass) × 100 [18,22]. The mean of three normalized readings will be considered for the analysis.

A digital algometer (Instrutherm™, model DD-200 (Instrutherm instrumentos de medição Ltda, São Paulo, Brazil) will be used to determine the pressure pain threshold. The participant will be positioned lying on one side on the cot and eight points will be marked on the knee(s): Point 1 - 2 cm below the medial edge of the patella; Point 2 - 2 cm below the lateral edge of the patella; Point 3 - 3 cm to the side of the midpoint of the lateral border of the patella; Point 4 - 2 cm above the lateral edge of the patella; Point 5 - 2 cm above the highest point of the upper edge of the patella; Point 6 - 2 cm above the medial edge of the patella; Point 7 - 3 cm to the side of the midpoint of the medial edge of the patella; and Point 8 - center of the patella [23]. A previously trained examiner will position the algometer perpendicular to each point and apply gradual pressure at a constant rate of approximately 0.5 kg/cm^2^/s. The points will receive pressure until the participant reports feeling pain and the value in kg/cm^2^ registered on the readout of the equipment will be recorded. The pressure pain threshold will be measured three times on each of the eight points, with the mean value of each point considered for the analysis.

A fleximeter (pendular goniometer; Sanny™(American Medical do Brasil Ltda, São Bernardo do Campo, Brazil) will be used for the evaluation of knee flexion. The participant will be placed in the prone position with the ankles off of the end of the cot. The fleximeter will be positioned on the lateral face of the ankle with the display turned toward the researcher. The researcher will stabilize the pelvis so that it does not move. For the evaluation of extension, the movement will begin with maximum knee flexion. The evaluation will be performed on both lower limbs. Two repetitions of each movement will be performed and the mean will be used for analysis [24].

The Timed up-and-go test (TUGT) is used to evaluate functional mobility by the time required for the participant to stand up from a chair, walk three meters, turn around, walk back to the chair, and sit down again. A chronometer (OREGON™ (Oregon Scientific Brasil LTDA – http://www.oregonscientific.com.br - Avenida Ibirapuera, 2907 – São Paulo/SP. Phone: 55 11 50952329) will be started after the verbal command ‘go’ and stopped when the participant has returned to the initial sitting position. The test will be performed twice. The first trial will be to familiarize the participant with the procedure and the time required to complete the second trial will be recorded. An armless chair with an adjustable height will be used to adjust the position of the knees of each individual at 90° flexion [25]. The following reference values will be considered: 10 seconds for healthy, independent adults with no risk of falls; 11 to 20 seconds for elderly individuals with disability or frailty; and more than 20 seconds indicates important mobility deficit and a risk of falls [26].

The Functional reach test (FRT) defines the maximum forward functional reach beyond one’s arm length while maintaining a fixed-foot base. A reach of less than 15 cm indicates frailty and an eminent risk of falls [27]. A metric tape will be attached to the wall parallel to the floor at the height of the acromion of each individual. The participant will be barefoot with feet parallel to the wall and near the beginning of the metric tape with the wrists in neutral position, elbows extended, and shoulder flexed at 90°. The individual will be instructed to lean the body forward without touching the tape. The movement of the wrist over the tape will be read. Three readings will be made, with the mean value used in the analysis.

#### Phototherapy

Phototherapy will be administered with a portable nine-diode cluster (PainAway™, Multi Radiance Medical, Solon, Ohio, United States), with one 905-nm diode (mean power: 1 mW; peak power: 10 W; spot size: 0.44 cm^2^), four 875-nm diodes (mean power of each diode: 17.5 mW; spot size: 1 cm^2^) and four 670-nm diodes (mean power of each diode: 15 mW; spot size: 1 cm^2^); frequency: 1000 Hz; 300-second irradiation time in each quadrant; total energy: 39.3 J per quadrant. The portable nine-diode cluster will be used overlapping three quadrants of the knee in random sequence: medial quadrant, lateral quadrant, and posterior quadrant. These quadrants were chosen to overlap the reference points employed in a study using a LLLT device with a smaller irradiation area [10].

#### Exercise program

The program will include three weekly 45 to 60-minute sessions over the course of eight weeks (24 sessions) and will be organized in three phases: 1) weeks one to two, improvements in balance and coordination, each exercise will have 30 repetitions and two sets; 2) weeks three to five, each exercise will have 20 repetitions and three sets; and 3) weeks six to eight, each exercise will have 20 repetitions and three sets [28].

Phase 1: 10 minutes of warm up on a treadmill; participant seated with weight on ankle extends the knee and performs internal and external rotation on each leg; in the supine position on a cot, from maximum knee flexion, the participant slowly performs maximum knee extension on each leg; in the standing position, the participant flexes the knee to 60° and performs knee extension; the participant walks along a three-meter line; if the participant steps outside the line, he/she must return to the initial position and in the standing position, the participant transfers weight from one leg to the other.

Phase 2: In the standing position, the participant flexes the knee to 60° and performs knee extension; the participant walks sideways crossing the legs to the right and to the left; in the standing position, the participant maintains his/her balance on a balance board; In the supine position on a cot, the participant performs maximum knee flexion on each leg;in the standing position, the participant positioned on a step flexes one knee and squats, followed by extension; the same is performed with the other leg.

Phase 3: The participant walks sideways crossing the legs to the right and to the left; in the standing position, the participant flexes the knee to 60° on each leg; in the standing position, the participant maintains his/her balance on a balance board; the participant is then asked to close his/her eyes; in the standing position, the participant holds the toes flexed for 1 to 2 seconds; participant seated with weight on ankle extends the knee and performs knee extension, holds for 3 to 4 seconds and returns slowly to flexion on each leg.

For exercises involving weight, the weight will be determined for each participant using the ten-maximum repetition test. New tests will be held at the beginning of each phase.

#### Data analysis

The sample size was calculated using the MVIC of the muscles as the outcome and an 80% power to detect a 20% improvement, with a 5% level of significance. Based on the study by Alfredo *et al.*[28], a minimum of 17 patients were determined for each group.

Primary (MVIC of the muscles (dynamometer) and stabilometry) and secondary (pain (digital algometer), range of motion (fleximeter), and quality of life (WHOQOL-BREF and WOMAC ) outcomes will be assessed.

Descriptive statistics will be used, and data will be summarized using mean, standard deviations, and percentiles. Normality of data distribution will be tested using the Kolmogorov-Smirnov test. Normal variables and comparison among three groups of the study will be assessed at baseline and at eight weeks follow-up using Analysis of Variance. Post-hoc tests will consist of the Turkey test for parametric variables, and the Dunn’s test for non-parametric variables, and the Dunn’s test for non-parametric variables. Data processing will be performed using the SPSS™ version 13.0 (Chicago, Illinois, United States) and the level of significance will be set to 5%.

The Cohen's d method will be used to determine the effect size. The interpretation of the values will be based on the classification established by Cohen: less than 0.2 (small effect); around 0.5 (moderate effect), up from 0.8 (large effect) [29].

## Discussion

The purpose of this randomized clinical trial is to analyze the effect of the incorporation of phototherapy into a therapeutic exercise program for OA of the knee. The study will support the practice based on evidence to the use of phototherapy in individuals with a diagnosis of osteoarthritis of the knee. Data will be published after the study is completed.

## Trial status

Patient recruitment is currently underway. Started on May 2014 and expected to finish on January 2015.

## Abbreviations

FRT: Functional reach test; LEFS: Lower extremity functional scale; LLLT: Low-level laser therapy; MVIC: Maximum voluntary isometric contraction; NSAIDs: Non-steroidal anti-inflammatory drugs; NRS: Numeric rating scale; OA: Osteoarthritis; ROM: Range of motion; TUGT: Timed up-and-go test; WOMAC: McMaster universities osteoarthritis.

## Competing interests

Professor Ernesto Cesar Pinto Leal-Junior receives research support from Multi Radiance Medical (Solon, OH - USA), a laser device manufacturer. The remaining authors declare that they have no competing interests.

## Authors’ contributions

CFC: conception and design, data collection and analysis, manuscript writing and final approval of the manuscript. ECPLJ: conception and design, financial support, manuscript writing, final approval of manuscript. ASB: data collection and analysis, critical revision and read and approved the final manuscript. FP: data collection and analysis, critical revision and read and approved the final manuscript. TOG: data collection and analysis, critical revision and read and approved the final manuscript. AVDF: data collection and analysis, critical revision and read and approved the final manuscript. DABG: conception and design, financial support, manuscript writing, final approval of manuscript. ARO: data collection and analysis, critical revision and read and approved the final manuscript. MF: data collection and analysis, critical revision and read and approved the final manuscript. MBSG: data collection and analysis, critical revision and read and approved the final manuscript. PTCC: conception and design, financial support, manuscript writing, final approval of manuscript. CAFPG: conception and design, financial support, manuscript writing, final approval of manuscript. All authors read and approved the final manuscript.

## Supplementary Material

Additional file 1 Flowchart of the study. Click here for file
