# Supplementary figures and images for: Effectiveness of phototherapy incorporated into an exercise program for osteoarthritis of the knee: study protocol for a randomized controlled trial
Source: Trials. 2014 Jun 11;15:221. doi: 10.1186/1745-6215-15-221 (PMC4229877; doi:10.1186/1745-6215-15-221)

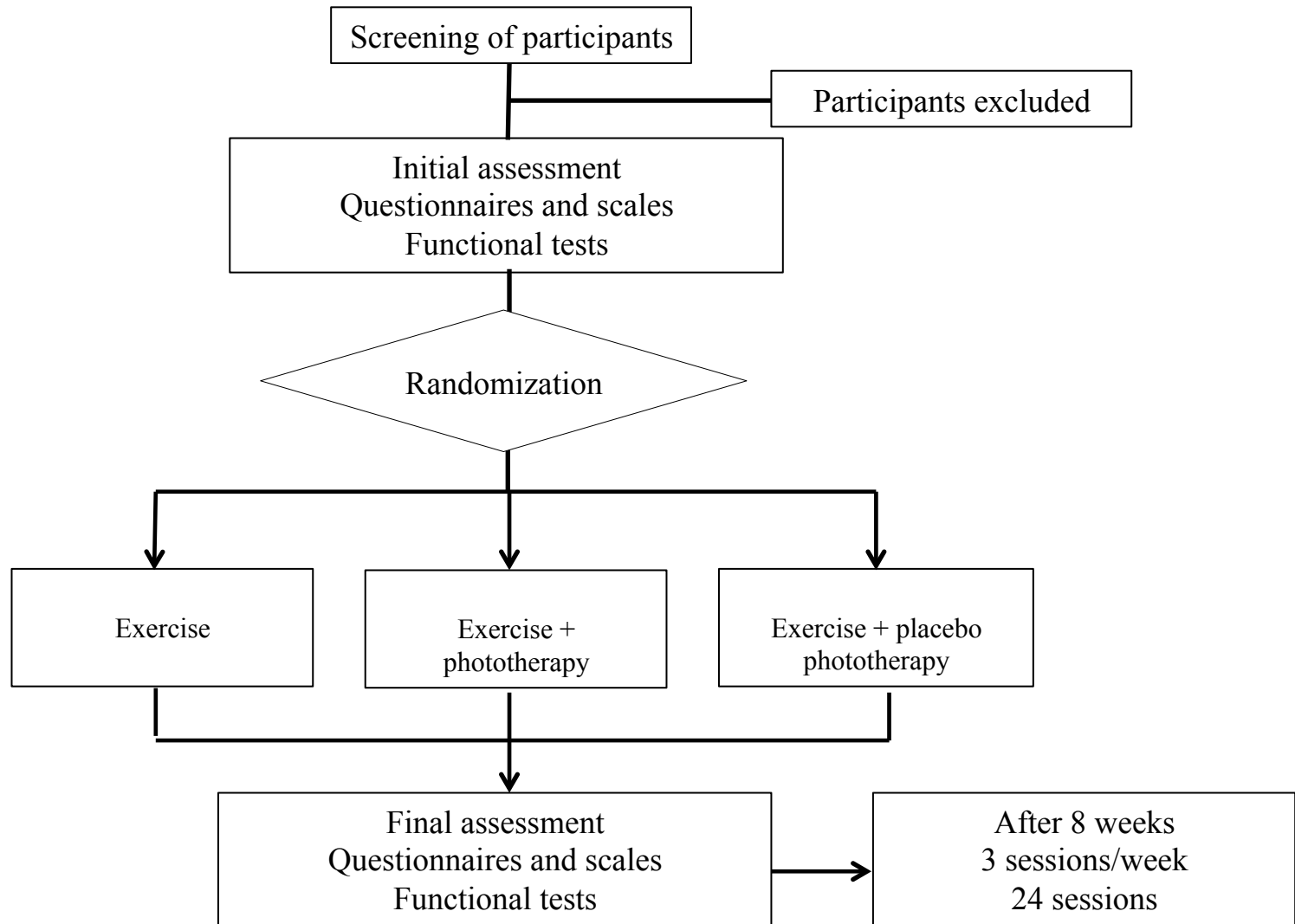

Supplement: Additional file 1 — Flowchart of the study. [file 1745-6215-15-221-S1.pdf]
